# Components of a new gene family of ferroxidases involved in virulence are functionally specialized in fungal dimorphism

**DOI:** 10.1038/s41598-018-26051-x

**Published:** 2018-05-16

**Authors:** María Isabel Navarro-Mendoza, Carlos Pérez-Arques, Laura Murcia, Pablo Martínez-García, Carlos Lax, Marta Sanchis, Javier Capilla, Francisco E. Nicolás, Victoriano Garre

**Affiliations:** 1 Departamento de Genética y Microbiología, Universidad de Murcia, 30100 Murcia, Spain; 2 Unidad de Microbiología, Universitat Rovira i Virgili. IISPV, Tarragona, Spain

## Abstract

Mucormycosis is an emerging angio-invasive infection caused by Mucorales that presents unacceptable mortality rates. Iron uptake has been related to mucormycosis, since serum iron availability predisposes the host to suffer this infection. In addition, iron uptake has been described as a limiting factor that determines virulence in other fungal infections, becoming a promising field to study virulence in Mucorales. Here, we identified a gene family of three ferroxidases in *Mucor circinelloides, fet3a, fet3b* and *fet3c*, which are overexpressed during infection in a mouse model for mucormycosis, and their expression *in vitro* is regulated by the availability of iron in the culture media and the dimorphic state. Thus, only *fet3a* is specifically expressed during yeast growth under anaerobic conditions, whereas *fet3b* and *fet3c* are specifically expressed in mycelium during aerobic growth. A deep genetic analysis revealed partially redundant roles of the three genes, showing a predominant role of *fet3c*, which is required for virulence during *in vivo* infections, and shared functional roles with *fet3b* and *fet3c* during vegetative growth in media with low iron concentration. These results represent the first described functional specialization of an iron uptake system during fungal dimorphism.

## Introduction

Mucormycosis is an emerging fungal infection caused by species of the order Mucorales that presents unacceptably high mortality rates. Although it was considered a rare infection in the past, its increasing diagnosis places this lethal infection as the third most common angio-invasive fungal infection after candidiasis and aspergillosis^1^. It has been traditionally described as a fungal infection affecting immunocrompromised patients suffering diabetes, cancer and organ transplantation^2^; however, the improvement in the diagnostic techniques has also shown the capacity of these organisms to produce local infections in immunocompetent patients, particularly after trauma^3,4^. Mortality rates of mucormycosis remain higher than 50% and reach up to 90% in disseminated infections as a direct consequence of lacking effective treatments and the intrinsic antifungal drug resistance of Mucorales^5–7^. In this regard, recent studies have revealed a new mechanism of antifungal drug resistance that is based in the RNAi mechanism of the human pathogen *Mucor circinelloides*. This novel mechanism produces epigenetically modified offspring repressing the expression of the gene *fkbp12*, which is the target of FK506^8,9^. In addition, Mucorales also present an outstanding natural resistance to antifungal drugs targeting the production of ergosterol, such us fluconazole, voriconazole, and itraconazole^10–12^, which is leading researchers to study different aspects of fungal physiology to find targets that could result in the development of new antifungal drugs^13^.

The knowledge about physiology of Mucorales and virulence factors associated to mucormycosis is still scarce compared to other well-known fungi like Ascomycota and Basidiomycota. Among the few factors that have been associated to virulence in pathogenic Mucorales^14^, their dimorphism or capability to alternate between yeast and mycelial forms represents a recent research field. *M*. *circinelloides* is a dimorphic fungus that propagates producing branching coenocytic hyphae in the presence of oxygen or spherical multipolar budding yeasts when deprived of oxygen^15^. The mycelial form of this opportunistic pathogen is linked to virulence through regulatory elements such as the calcineurin pathway and structural processes like intracellular cargo transport, which have been proven essential for the transition from yeast to mycelium and, consequently, for pathogenesis^13,16,17^. Besides dimorphism, iron availability and uptake have become the most promising virulence factors that could be translated into new targets for antifungal drugs^18–21^. Indeed, the most relevant factor that currently has been successfully associated to the susceptibility of patients to suffer mucormycosis is the elevated available serum iron, a condition that can be found in patients suffering hyperglycemia and diabetic ketoacidosis^22^. Under these conditions, the abnormal low pH destabilizes the iron chelating systems of the host, increasing the amount of free ferric ion (Fe^3+^). This abnormal condition supposes an advantage for fungal pathogens which have developed a three-component iron reduction system for the uptake of Fe^3+^ ^23^. The first component of this system is a plasma membrane metalloreductase or ferric reductase (encoded by *fre* genes) which reduces Fe^3+^ to Fe^2+^ ^24^. The ferrous ion Fe^2+^ is then oxidized by an iron transport multicopper ferroxidase (encoded by *fet3* genes) to Fe^3+^ ^25^, which can be loaded into the third component of the system, a high-affinity iron permease (encoded by *ftr1* genes) that finally allows its transport inside the cell^26^. The role of the FRE family of plasma membrane reductases in iron uptake was studied in *Saccharomyces cerevisiae*, in which several homologous metalloreductases were described with a redundant function that confers the ability to utilize iron from a variety of sources^27^. These iron reductases have been associated to virulence in the human pathogen *Cryptococcus neoformans*, in which mutants lacking the gene *FRE2* showed attenuated virulence. This phenotype was linked to the growth defects observed in these strains as a consequence of their inability to use iron from heme and transferrin sources of the host^28^. Similarly, the role of the multicopper ferroxidase Cfo1, which is an ortholog of *S. cerevisiae* ferroxidase FET3^25^, has also been linked to virulence in *C. neoformans*. A mutant lacking both *CFO1* and *FRE2* genes showed a more pronounced growth defect, indicating that both components participate in the high-affinity uptake system^28^. Regarding Mucorales, only the high-affinity iron permease Ftr1 has been characterized and associated to virulence in *Rhizopus oryzae*. Specifically, mutants in *ftr1* are compromised in their ability to acquire iron during *in vitro* culturing and present reduced virulence during *in vivo* infections in mouse models^20,21^. These studies indicate a crucial role of the high-affinity iron uptake system in the development of mucormycosis, correlating the susceptibility of patients presenting unbalanced levels of available iron to this infection.

In sight of the promising results that previous studies have established between the high-affinity iron uptake system and virulence in mucormycosis, the main objective of this work was to identify genes involved in iron metabolism, and subsequently study their functional role under conditions related to virulence. In this sense, we searched the genome of the genetic fungal model *M. circinelloides* for genes encoding proteins with high similarity to FET3 of *S. cerevisiae*^25^, which is a partner of the permease FTR1 in the high-affinity iron uptake system and a putative candidate to play an important role in mucormycosis. Our results showed the existence of a gene family that encompassed three genes encoding putative ferroxidases similar to *S. cerevisiae* FET3^25^. Expression of these genes has been studied in various conditions mimicking the host environment, in both morphological states of *M. circinelloides* and in lung tissue of infected mice, revealing for the first time the specialization among a ferroxidase family in different developmental stages of this dimorphic fungus. Furthermore, the generation of single and double mutants in this gene family implicated ferroxidases both in vegetative growth on media with low iron concentration and virulence in a mammalian model.

## Results

### *M. circinelloides* genome encodes three putative ferroxidases overexpressed during mouse infection

**Figure 1:**
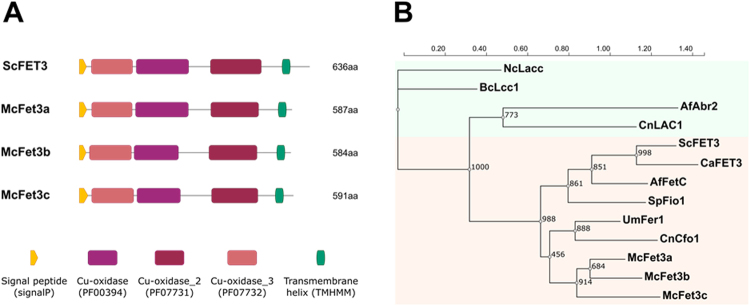
Conservation analysis of the proteins Fet3a, Fet3b and Fet3c. (**A**) Schematic comparison of *M. circinelloides* Fet3 proteins (shown as McFet3a, McFet3b and McFet3c) with *S. cerevisiae* FET3 (ScFET3), depicting protein domain architecture, signal peptide and transmembrane helixes. (**B**) Phylogenetic analysis of well-characterized fungal ferroxidases (pink background) and laccases (green background) and their relationship with *M. circinelloides* putative ferroxidases, showing support values for each node. Protein names and ID numbers are listed on Supplementary Table S1.

The genome of *M. circinelloides* (CBS277.49, v2.0; http://genome.jgi-psf.org/Mucci2/Mucci2.home.html) was searched by BLAST to identify orthologous genes of the *S. cerevisiae* ferroxidase *FET3*^25^ (ID: YMR058W-t26_1; FugiDB.org), resulting in the identification of three putative ferroxidase/multicopper oxidases in this fungus. These three candidates contained a protein sequence harboring typical domains of ferroxidase/multicopper oxidase enzymes (hereinafter ferroxidases), including a Cu-oxidase_3 Multicopper oxidase (PF07732/ IPR011707), a Cu-oxidase Multicopper oxidase (PF 00394/IPR001117) and a Cu-oxidase_2 Multicopper oxidase domains (PF07731/IPR011706) (Fig. 1A). In addition to these domains, a putative signal peptide and a transmembrane region presented the same position and structure in the three putative ferroxidases, suggesting that they are secreted proteins bound to the plasmatic membrane (Fig. 1A). This elevated similarity in the structure and the exceptionally high sequence identity (95%) suggested that they could be members of the same gene family. To test this hypothesis, a phylogenetic study was performed comparing a set of known fungal ferroxidases, *M. circinelloides* ferroxidases and fungal laccases (Supplementary Table S1) that shared similar domain structure but different function (Fig. 1B). This analysis showed a close clustering of the three *M. circinelloides* proteins in the same branch as other well-characterized fungal ferroxidases, including *S. cerevisiae* ferroxidase FET3. Thus, based in the high similarity between the three *M. circinelloides* ferroxidases and according to their close phylogenetic proximity to *S. cerevisiae* FET3, they were named *fet3a* (ID 187130), *fet3b* (ID 50174) and *fet3c* (ID 91148) (Fig. 1).

**Figure 2:**
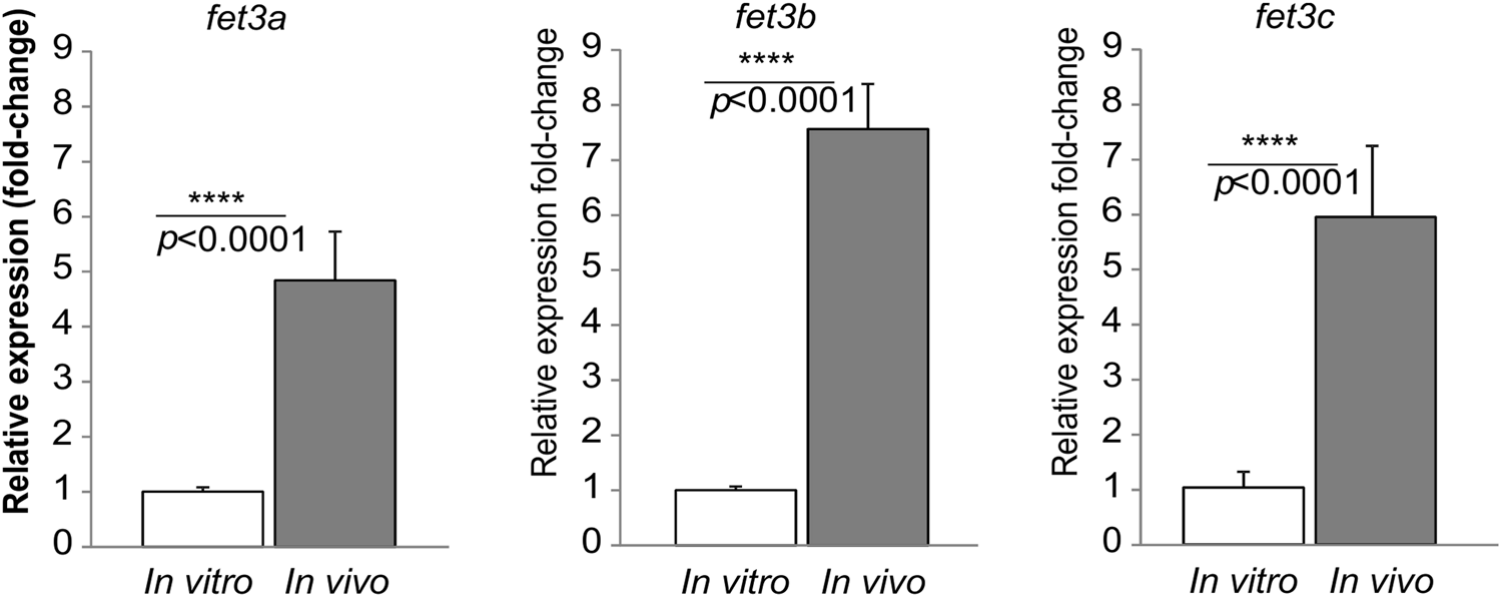
Differential expression of *fet3a, fet3b* and *fet3c* genes in infected mice and mycelia. The bars indicate the relative gene expression (mean ± s.d.) of the target genes in infected mice (*in vivo*, black bars, n = 5) and mycelia (*in vitro*, white bars, n = 5) compared to mycelia, after normalization with the 18S rRNA expression. Data were analyzed using a two-tailed unpaired t test (****p < 0.0001, T-test).

The expression of *M. circinelloides* ferroxidases was studied during infection in OF-1 mice, since iron uptake is a crucial stage at the onset of the infection, and *in vivo* regulation in a host model suggests a functional role in the pathogenesis of mucormycosis^29^. The expression of *fet3a*, *fet3b* and *fet3c* was analyzed by reverse transcription quantitative PCR (RT-qPCR) in total RNA extracted from lung tissues of mice infected intravenously with 10^6^ spores^13^ at day two post-infection, compared to total RNA isolated from mycelia grown *in vitro* on solid rich medium YPG. The day two post-infection was chosen because of the high fungal burden observed at that moment in mice infected with *M. circinelloides*^13^. The RT-qPCR assays showed a significantly increased expression of the genes *fet3a*, *fet3b* and *fet3c* during infection in mice (n = 5, *p* < 0.0001 unpaired t-test) (Fig. 2). These assays showed a 4.8-, 7.7- and 5.9-fold overexpression of the genes *fet3a*, *fet3b* and *fet3c*, respectively during mice infection compared to their expression when the spores were grown in rich medium [median (25th percentile, 75th percentile) = 4.71 (4.37, 5.39); 7.23 (6.96, 8.48); 6.26 (4.98, 6.46) for *fet3a, fet3b* and *fet3c*, respectively]. These results demonstrated that the three ferroxidase genes found in *M. circinelloides* are all expressed during *in vivo* infections and strongly induced when compared to their expression *in vitro*, likely due to the low availability of iron inside the host.

### Expression of the genes *fet3a*, *fet3b* and *fet3c* is iron responsive

**Figure 3:**
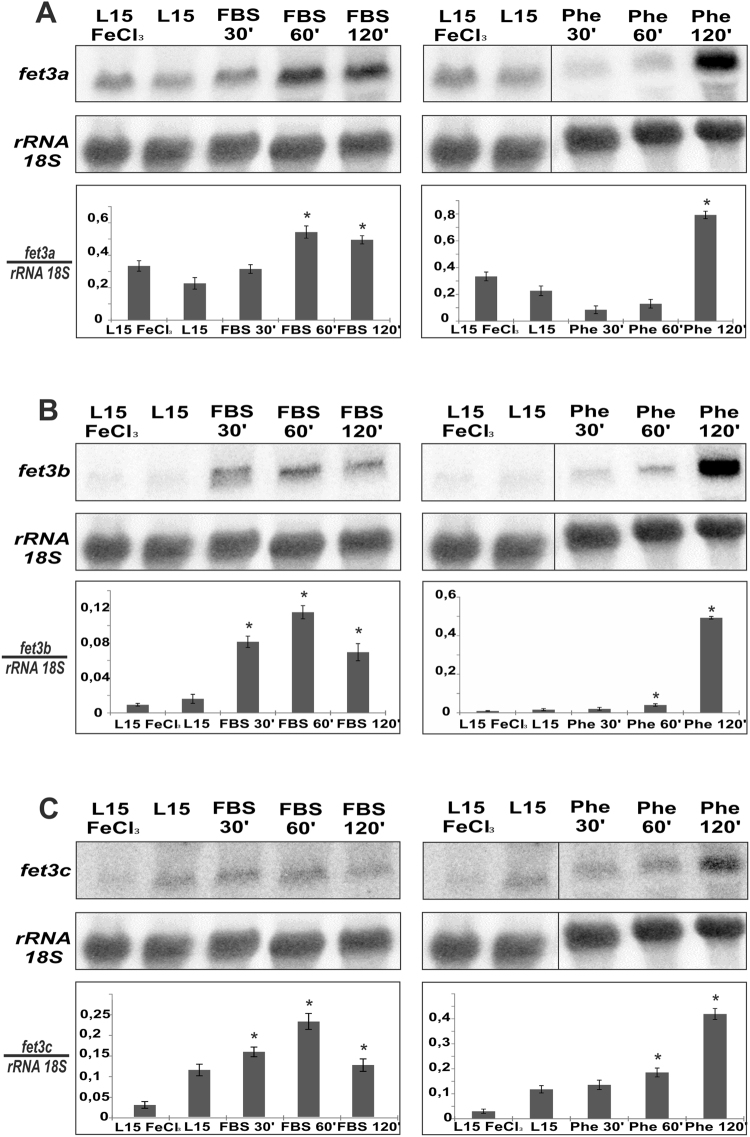
Induction of the genes *fet3a, fet3b* and *fet3c* by low iron availability. Upper panels show levels of *fet3a, fet3b* and *fet3c* mRNAs (**A**,**B** and **C**, respectively). Total RNA was extracted from mycelia of the wild type strain R7B grown in liquid culture for 24 hours in YNB pH 3.2, then the mycelia was washed and grown in either L15 or L15 + FeCl_3_ 350 μM. To analyze expression of the *fet3* genes in response to iron limitation, the mycelia grown in L15 were washed and transferred to an iron depleted media (with FBS or 1,10-phenanthroline) and samples were taken at 30 min, 60 min and 120 min. Middle panels show mRNA loading controls, which were performed by re-probing the membranes with a rRNA 18S probe. Lower panels show relative accumulation of *fet3a, fet3b* and *fet3c* mRNAs (A, B and C, respectively) by normalization with the 18S rRNA signals. Significant differences respect to control L15 medium are indicated with an *(*p* < 0.01, unpaired t-test). The cropped blots are displayed in the main figure, the black lines surrounding blots indicate the cropping lines. The scanned full blots are presented in Supplementary Fig. 2.

In *C. neoformans* and *S. cerevisiae* the expression of FRE genes have been found to be responsive to the amount of iron available in the growth media^28^. Similarly, *FET3* gene of *S. cerevisiae* is expressed under low iron availability through the activation mediated by the transcriptional factors Aft1p and Aft2p^30^. These results indicated that the high-affinity iron uptake is a finely regulated system that is expressed only when the amount of available iron is not enough to sustain regular growth in yeasts. In order to extend this analysis to Mucorales, the expression of the genes *fet3a*, *fet3b* and *fet3c* was analyzed in *M. circinelloides* under four different liquid growth conditions: high iron medium, iron-depleted medium mimicking iron chelation in the host, iron-depleted medium by a synthetic iron chelator and control non-modified medium (see Materials and Methods). Both the synthetic and host-mimicking assays were time-course tested at three different times (30, 60 and 120 minutes) in order to analyze the induction kinetics of the three genes. Under these growth conditions, the expression of *fet3a*, *fet3b* and *fet3c* was analyzed by northern blot hybridizations, which showed a significant increase in the mRNA levels of all three genes as a consequence of the lack of available iron provoked by both natural and synthetic chelating agents (*p* < 0.01, unpaired t-test) (Fig. 3). The gene *fet3a* showed a basal expression in L15 medium that was not reduced in high iron medium (Fig. 3A), which was almost undetectable for the gene *fet3b* in both conditions (Fig. 3B). On the contrary, gene *fet3c* showed a four-fold increased expression in L15 relative to high iron medium (Fig. 3C). Regarding host-mimicking medium, all three genes showed a rapid increase in their mRNA levels with a peak at 60 minutes, when compared to the levels in control L15 medium. Genes *fet3a* and *fet3b* showed a significant difference (*p* < 0.01 unpaired t-test, 2.4-fold and 6.5-fold, respectively) compared to the subtle increase observed in *fet3c* (*p* < 0.01 unpaired t-test, 1.4-fold). On the other hand, in the presence of a synthetic iron chelator, *fet3a*, *fet3b* and *fet3c* presented a delayed response with a peak at 120 minutes (4-fold, 25-fold and 4.5-fold, respectively), compared to the expression detected in control L15 medium, though the induction at this point was higher when compared to signals detected in the host-mimicking medium at 60 minutes (Fig. 3). The ferroxidase gene family of *M. circinelloides* is thereby induced by low iron availability, both with natural and artificial iron chelation.

### Generation of single and double mutants in the genes *fet3a*, *fet3b* and *fet3c*

**Figure 4:**
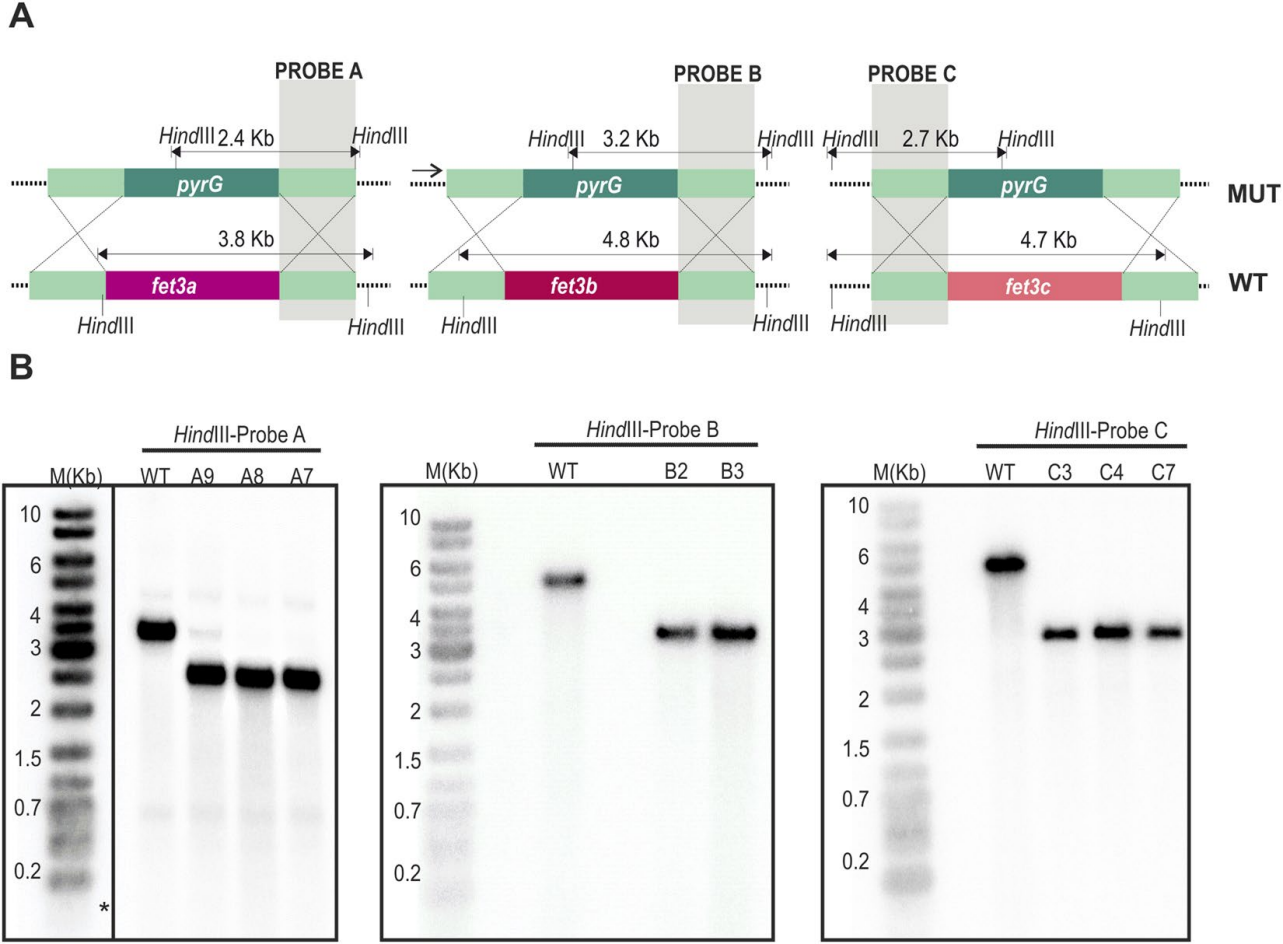
Disruption of genes *fet3a, fet3b* and *fet3c*. (**A**) Schematic representation of wild-type and mutant loci after homologous recombination with the disruption fragments of genes *fet3a* (left), *fet3b* (middle) and *fet3c* (right). The position of the probes used (A, B and C) and the expected sizes of the restriction fragments are indicated. Dashed lines are genomic sequences not included in the disruption fragment. (**B**) Southern blot analysis of the wild-type recipient strain (WT) and transformants obtained with the disruption fragments after ten vegetative cycles in selective medium. Genomic DNA (1 μg) was digested with *Hind*III and hybridized with probes A, B and C, which recognized wild type and disrupted alleles, but could discriminate between them. The positions and sizes of the GeneRuler DNA ladder mixture (M) (Fermentas) are indicated. The cropped blots are displayed in the main figure, the black lines surrounding blots indicate the cropping lines. The scanned full blots are presented in Supplementary Fig. 3. Asterisk (*) indicated that marker M(Kb) was revealed after a second re-hibridization of the same membrane.

The expression analyses of the genes *fet3a*, *fet3b* and *fet3c* indicated that *M. circinelloides* ferroxidases are required both under low concentration of available iron and during infection in a mouse model. Together, these two results suggested an active role of *fet3* genes in the iron uptake during the progression of mucormycosis. Thus, the role of these three genes was studied through the generation of the corresponding single and double mutants by homologous recombination (see Materials and Methods). Deletion mutants for all three genes could not be generated since there are only two auxotrophic selectable markers available in *M. circinelloides*^31^. In addition, *M. circinelloides* produces multinucleated spores that can contain a mixture of mutant and wild type nuclei after transformation with disruption cassettes. Thus, the correct disruption and the homokaryosis for the mutant allele were also analyzed by Southern blot hybridization in all the isolates that were selected after the PCR screenings (Fig. 4 and Supplementary Table S3). Regarding the disruption of *fet3a*, three independent transformants were positive in the PCR screening (Supplementary Fig. S1; A7, A8 and A9), and two of them were homokaryons confirmed by Southern blot hybridization (Fig. 4; A7 and A8). Similarly, two and three independent transformants were confirmed for homokaryotic disruption of *fet3b* (Fig. 4; B2 and B3) and *fet3c* (Fig. 4; C3, C4 and C7), respectively.

**Figure 5:**
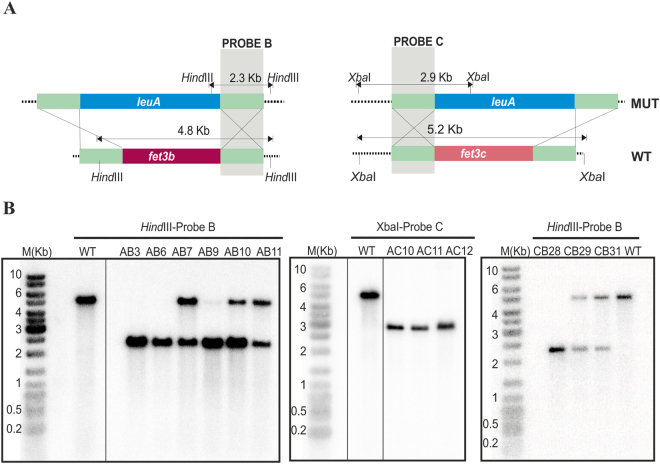
Generation of double deletion mutants Δ*fet3a*/Δ*fet3b*, Δ*fet3c/*Δ*fet3b* and Δ*fet3a/*Δ*fet3c*. (**A**) Schematic representation of wild type and mutant loci after homologous recombination with the disruption fragments of genes *fet3b* (left) and *fet3c* (right). The position of the probes used (B and C) and the expected sizes of the restriction fragments are indicated. Dashed lines, sequences not included in the disruption fragment. (**B**) Southern blot analysis of the wild-type recipient strain (WT) and transformants obtained with the disruption fragments after ten vegetative cycles in selective medium. Genomic DNA (1 μg) was digested with *Hind*III (transformants for *fet3b*, left and right) or *Xba*I (transformants for *fet3c*, middle) and hybridized with probes B and C, which recognized wild-type and disrupted alleles, but could discriminate between them. The positions and sizes of the GeneRuler DNA ladder mixture (M) (Fermentas) are indicated. The cropped blots are displayed in the main figure, the black lines surrounding blots indicate the cropping lines. The scanned full blots are presented in Supplementary Fig. 3.

Once the single mutants Δ*fet3a*, Δ*fet3b* and Δ*fet3c* were generated, double mutations were carried out using new disrupting cassettes containing the gene *leuA*, which can complement the leucine auxotrophy present in these strains (Fig. 5, upper panels). The mutant Δ*fet3a* was used as the recipient strain for genetic transformation with cassettes containing flanking sequences of the genes *fet3b* and *fet3c* to generate the double deletion mutants Δ*fet3a/*Δ*fet3b* and Δ*fet3a/*Δ*fet3c*, respectively (Fig. 5). For the double mutant Δ*fet3a/*Δ*fet3b*, six PCR positive transformants (Supplementary Fig. S1) were analyzed by Southern blot hybridization and two of them were confirmed for homokaryosis (Fig. 5, AB3 and AB6). For the double mutant Δ*fet3a/*Δ*fet3c*, the three PCR positive transformants were confirmed for homokaryosis (Supplementary Fig. S1 and Fig. 5; AC10, AC11 and AC12). Equally, the mutant Δ*fet3c* was used as the recipient strain for the genetic transformation with a cassette containing *leuA* gene and flanking sequences of the gene *fet3b*, which generated the double mutant Δ*fet3c*/Δ*fet3b* (Fig. 5). One out of three PCR positive transformants was confirmed for homokaryosis by Southern blot (Supplementary Fig. S1 and Fig. 5, transformant CB28).

### Role of *fet3* genes under low iron availability

**Figure 6:**
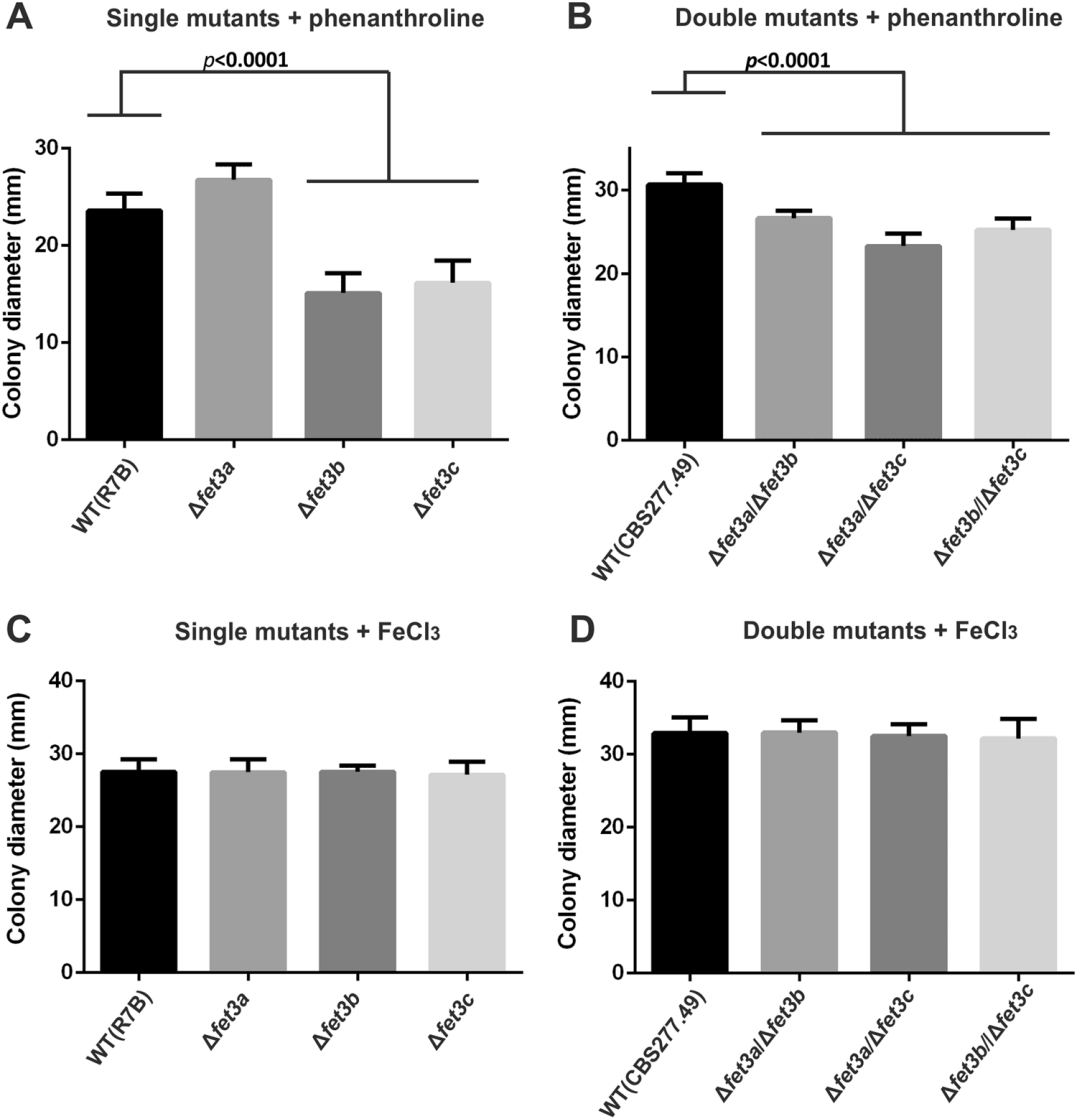
Growth defects of single and double deletion mutants in the genes *fet3a, fet3b* and *fet3c*. The diameter of ten independent colonies was measured from single deletion mutants Δ*fet3a*, Δ*fet3b and* Δ*fet3c* in (**A**), and double deletion mutants Δ*fet3a*/Δ*fet3b*, Δ*fet3c/*Δ*fet3b* and Δ*fet3a/*Δ*fet3c* in (**B**), and compared to their corresponding wild-type control strains. Cultures were grown in solid minimal media YNB pH 4.5 supplemented with 50 μM of 1,10-Phenanthroline for 48 hours. In (**C**) and (**D**) single and double deletion mutants (respectively) and wild-type strains were grown as described above, but using media without 1,10-Phenanthroline and supplemented with FeCl_3_ 350 µM.

The expression of genes *fet3a*, *fet3b* and *fet3c* is strongly induced when iron is not available (Fig. 3), suggesting a functional role of their encoded proteins in iron metabolism during regular development. Thus, after the generation of single and double mutants, their growth pattern was tested in media with and without available iron. The differences in growth rate of single and double mutants in ferroxidase genes are compared with their proper control strains, i.e. that had the same auxotrophic phenotype, considering that differences in *leuA* or *pyrG* content may disguise the phenotype of the *fet3* gene deletion. Consequently, the phenotype of single mutants which are auxotrophic for leucine were compared to the wild-type strain R7B (*pyrG*^+^*/leuA*^*−*^), while prototrophic double mutants were compared to the wild-type strain CBS277.49 (*pyrG*^+^*/leuA*^+^). The three single mutants Δ*fet3a*, Δ*fet3b* and Δ*fet3c* were grown in solid YNB medium supplemented with leucine (20 mg/l) and phenanthroline (50 μM), and compared to their control strain (R7B). At 48 h colony diameters were determined for 10 independent replicates of each strain (Fig. 6A). Both Δ*fet3b* and Δ*fet3c* mutants showed a significant reduction in their growth (p < 0.0001, unpaired t-test) (Fig. 6A), while mutant Δ*fet3a* growth was not affected in iron-depleted medium. Regarding the double mutants Δ*fet3a/*Δ*fet3b*, Δ*fet3a/*Δ*fet3c* and Δ*fet3c*/Δ*fet3b*, their growth was analyzed on solid YNB medium supplemented with phenanthroline (50 μM), and compared to the control wild-type strain CBS277.49). Measures were taken similarly to the single mutants and, in this case, all three double mutants showed a significant reduction in their growth (p < 0.0001, unpaired t-test) (Fig. 6B). All the mutant strains presented similar growth to the wild type strains when they were grown on iron-rich solid YNB medium (supplemented with 350 μM de FeCl_3_) (Fig. 6C and D). These results implied that this gene family of ferroxidases is involved in iron metabolism, considering that deletion in more than one ferroxidase gene results in defects in growth in iron depleted conditions.

### The gene *fet3a* is specifically expressed during yeast growth

**Figure 7:**
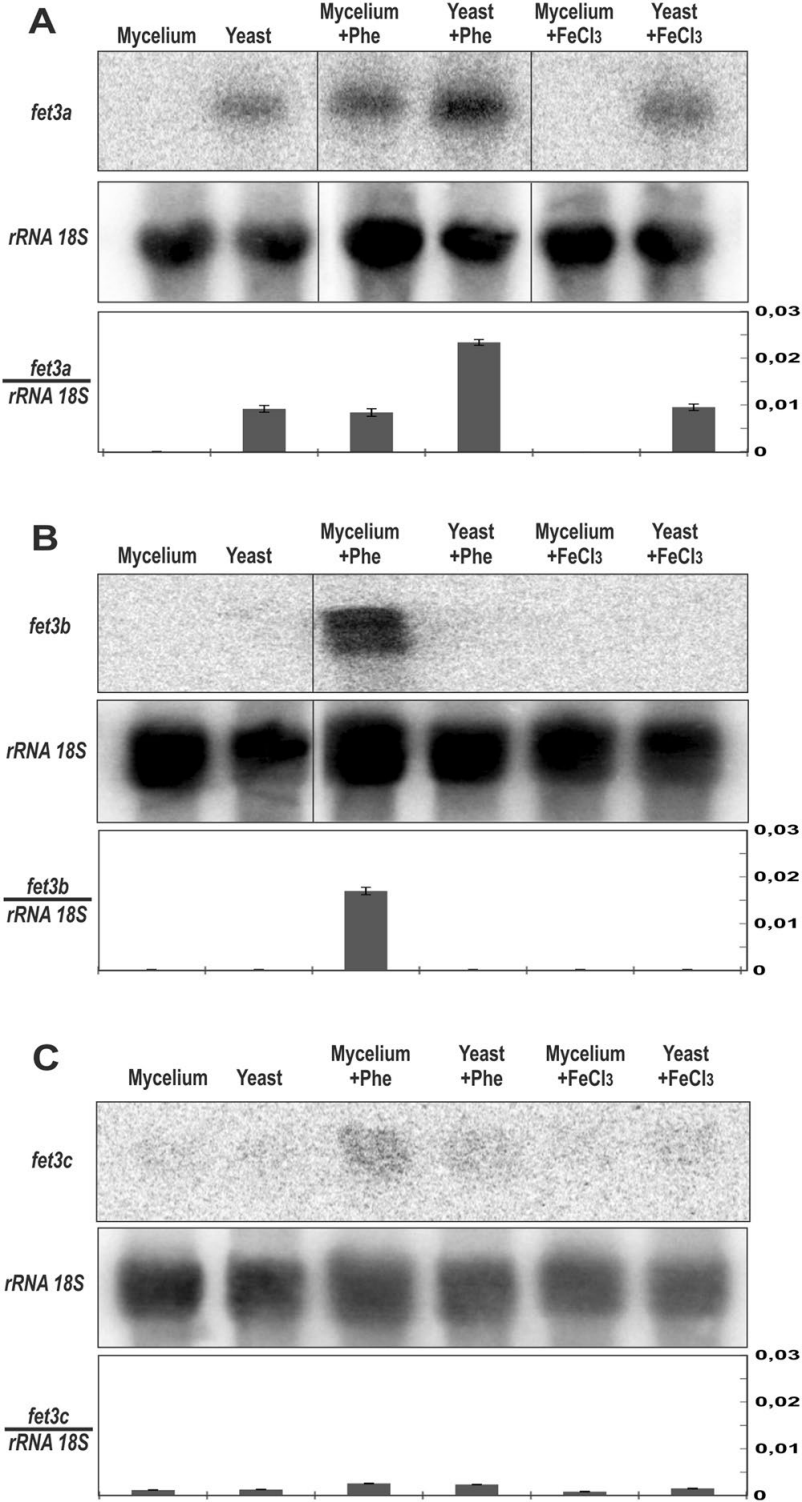
Differential expression of the genes *fet3a, fet3b* and *fet3c* during dimorphic development. Upper panels show levels of *fet3a, fet3b* and *fet3c* mRNAs (**A**,**B** and **C**, respectively) during yeast and mycelial growth. Two cultures of 50 ml of YPG 4.5 inoculated with 10^6^ spores/ml were grown in a tightly closed 50-ml conical tube for 15 hours to induce yeast growth. Next, one of the tubes was opened and transferred to an open 500 ml flask in which it was shaken for 2 hours to promote the mycelial growth. These two cultures were repeated adding either 1,10-phenanthroline 10 μM or FeCl_3_ 350 μM. Middle panels show mRNA loading controls, which were performed by re-probing the membranes with a rRNA 18S probe. Lower panels show relative expression of the genes *fet3a, fet3b* and *fet3c* mRNAs (**A**,**B** and **C**, respectively) by normalization with the 18S rRNA signals. The cropped blots are displayed in the main figure, the black lines surrounding blots indicate the cropping lines. The scanned full blots are presented in Supplementary Fig. 4.

Previous results showed no effects of *fet3a* disruption in the mycelial growth on media with low available iron, suggesting that either this gene is not functional or it is playing its functional role in a different stage of the dimorphic development of *M. circinelloides*. Thus, in order to test the hypothetical role of the three ferroxidases in the development of *M. circinelloides*, the expression of the three genes was analyzed in yeast and mycelial forms. Yeast growth was induced by inoculating 1 × 10^6^ spores/ml in a tube completely filled with rich liquid YPG medium and cultured overnight keeping the tube absolutely closed for anaerobic conditions. Induction of mycelial growth was obtained by transferring overnight grown yeasts to a large Erlenmeyer flask and incubating another 2 hours with vigorous shaking (250 rpm), which rapidly induced the polar growth of the yeasts. In addition, these two yeast/mycelium cultures were replicated in another two conditions: with low available iron through the addition of phenanthroline (10 μM) or with exceeding iron by adding FeCl_3_ (350 μM). Total RNA from these six samples was purified and the expression of the genes analyzed by northern blot hybridization (differences were considered significant with *p* < 0.01, unpaired t-test) (Fig. 7). In yeast form, only expression of *fet3a* was significantly increased, being induced by iron-depleted conditions (Fig. 7A). Conversely, *fet3b* was undetectable in the yeast form independently of iron levels but it was strongly induced by low iron availability (phenanthroline) in the mycelial form (Fig. 7B); while *fet3c* presented a low expression level in both forms that was induced by low available iron (Fig. 7C). These results indicated a functional specialization in the ferroxidases gene family of *M. circinelloides* through the dimorphic development of this fungus.

### Role of the genes *fet3a*, *fet3b* and *fet3c* in virulence of *M. circinelloides*

**Figure 8:**
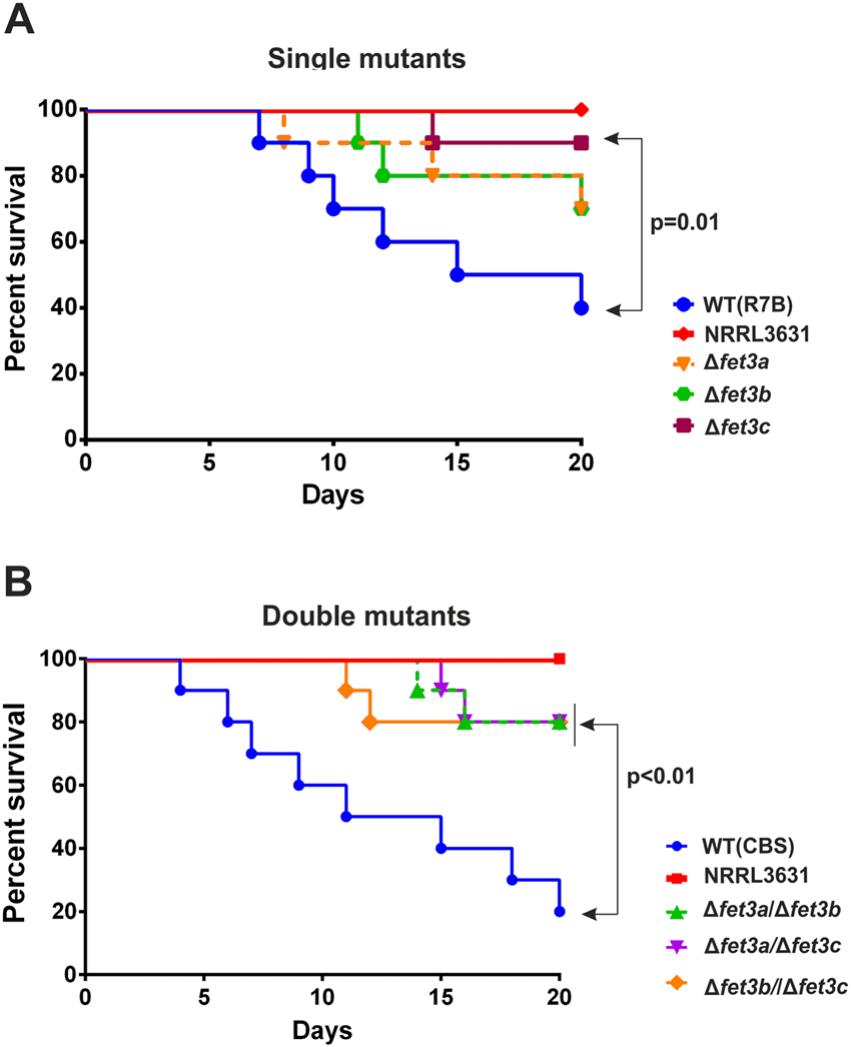
Virulence test of single and double deletion mutants in the ferroxidase genes in a murine host model. (**A**) Virulence assays using spores of the wild type strain and the single deletion mutants Δ*fet3a*, Δ*fet3b and* Δ*fet3c*. (**B**) Virulence assays using spores of the wild type strain and the double deletion mutants Δ*fet3a*/Δ*fet3b*, Δ*fet3c/*Δ*fet3b* and Δ*fet3a/*Δ*fet3c*. Immunosuppressed mice were injected with 1 × 10^6^ sporangiospores of each strain.

The induction of *fet3* genes during mouse infection and their role in vegetative growth under low-iron availability suggested a relevant function of ferroxidases in mucormycosis. Thus, the virulence potential of mutant strains was compared to the wild type in a murine model of mucormycosis. Virulence of single *fet3* mutants during infection in OF-1 mice was compared to virulent control strains R7B (*pyrG*^+^, *leuA*^*−*^), assuring that virulence differences correspond to ferroxidase deletion and not to leucine auxotrophy. Similarly, virulence of double *fet3* mutants was compared to the wild-type virulent strain CBS277.49 (*pyrG*^+^, *leuA*^+^). The avirulent wild-type strain NRRL3631 (*pyrG*+, *leuA*+) was used in every virulence assay as a control of the infection method^13,32^. Regarding the single deletion mutants, the three strains showed reduced virulence relative to the virulent control (mortalities of 60% virulent strain vs 30% Δ*fet3a*, 30% Δ*fet3b* and 10% Δ*fet3c* by the end of the experiment) (Fig. 8A). However, the reduction in the mortality rates was only statistically significant in the case of the mutant Δ*fet3c* (p = 0.01, Log-rank [Mantel-Cox] test), suggesting that gene *fet3c* plays the main role in iron uptake during the infection process. On the other hand, the three double mutants showed a uniform reduction in virulence relative to the control CBS277.49 (mortality of 80% for control strain vs 20% for double mutants) with statistical significance in the three comparisons (p < 0.01, Log-rank [Mantel-Cox] test) (Fig. 8B). These results suggested that *fet3c* plays the main role in virulence of *M. circinelloides*, but genes *fet3a* and *fet3b* might also participate by complementing the action of *fet3c*.

## Discussion

Iron participates in numerous metabolic processes as a vital element in most organisms. However, this element is not easily accessible from the environment, since it is found as insoluble ferric hydroxides, driving the evolution of specific uptake systems in fungi^23^. In addition, pathogens specialized in mammalian hosts have to confront specific chelating systems that have been designed to limit the amount of available iron^33^. Among these pathogens, fungi infecting human hosts employ several iron acquisition mechanisms that usually are essential for the infection, and consequently important factors for their virulence. This is the case of the production and uptake of siderophores in the invasive fungus *Aspergillus fumigatus*^34–36^, or the high-affinity iron uptake system of *C. neoformans* and *Candida albicans*^37,38^. In Mucorales the link between the available iron in the host and virulence is even more intriguing, since high available iron in patients is one of the few factors associated to their susceptibility to develop mucormycosis^29^. Besides this preference for iron-rich environments, the dimorphic yeast/mycelium development of Mucorales also has been related to virulence in several studies, since infections using the yeast form do not develop mucormycosis^16,17^. Mucorales are opportunistic pathogens in which the study of classic virulence factors has not yet identified a clear mechanism to explain the virulence of these organisms. In consequence, the requirement for iron in mucormycosis and the determination of the yeast form are the only two weaknesses associated to Mucorales, which are being studied as promising research fields for the discovery of new targets that can be used for the treatment of this fungal infection. Here, we report for the first time a link between these two weaknesses of mucormycosis: a detailed genetic analysis of the three members of a ferroxidase gene family that showed a specific expression pattern in the avirulent yeast and virulent mycelial forms. Moreover, this genetic approach dissected the role of each gene in the virulence of *M. circinelloides*, finding a partially redundant and pivotal role of the three genes during the infection process.

The three members of the ferroxidase gene family of *M. circinelloides* showed a high sequence similarity, suggesting that they are the result of several duplication events in an ancestral gene, an evolutionary process that is characteristic of Mucorales^39^. Subsequently, the members of the ferroxidase family may have undergone a process of subfunctionalization, which is initially associated with distinct expression patterns under different environmental changes^40^. Thus, the expression of the three ferroxidases in yeasts and mycelium revealed a specific response of the gene *fet3a* during yeast growth under anaerobic conditions, both under regular and low iron levels; whereas *fet3b* and *fet3c* presented an undetectable or very low expression in these conditions, respectively. In mature mycelium, the three genes were induced by low iron levels. These results indicated a functional specialization of the ferroxidases gene family in *M. circinelloides*, in which gene *fet3a* might be involved in iron uptake during yeast growth, whereas the three genes share a redundant function during mycelial growth under low iron levels. This functional specialization in gene families of *M. circinelloides* has been previously observed in several processes in this fungus, such as in the RNA interference mechanism, where *rdrp1, rdrp2* and *rdrp3* genes coding for RNA-dependent RNA polymerases presents distinct functional roles in the different silencing pathways^8,41,42^. The dimorphism between mycelium and yeast is critical for pathogenesis and virulence in many fungi; however, among the different dimorphic pathogenic fungi, the virulent form is not always the same. For instance, in thermally dimorphic fungi like *Histoplasma capsulatum* and *Coccidioides* spp. the virulent form is the yeast^43^, whereas *M. circinelloides* is unable to infect as a yeast and *C. albicans* switches to hyphal growth to invade tissue^16,17,44^. This divergence between species indicates that the dimorphic forms of yeast or hyphae are not the sole reason for virulence and there must be other aspects responsible for virulence differences, like structure and composition of the cell wall. Here, we showed how *M. circinelloides* ferroxidase genes *fet3a, fet3b* and *fet3c* are differentially expressed in yeast and hyphae, which could represent a subfunctionalization of the high-affinity iron uptake system in the two dimorphic states of this fungus.

The *in vivo* expression studies of this ferroxidase family revealed that all three genes are induced in lung tissue of infected mice (Fig. 2), probably as a consequence of the low iron availability in the host^21^. In addition, these results suggest that the three ferroxidases described here could have a prominent role in the virulence of *M. circinelloides*, since iron acquisition is essential for the development of mucormycosis^29^. Their role in virulence was analyzed through a detailed genetic dissection that investigated the function of each gene and the redundancies between them in *M. circinelloides*. Thus, the analysis of single deletion mutants indicated that *fet3b* and *fet3c* play the main role in the acquisition of iron during mycelial growth, which further supported their involvement in virulence, though only the mutant in the gene *fet3c* presented a statistically significant reduced virulence. These results indicated that the principal ferroxidase acting during *in vivo* infection of mammalian hosts is encoded by the gene *fet3c* in the fungus *M. circinelloides*. However, the three double mutants showed a statistically significant reduced virulence compared to the control strain, including the mutant Δ*fet3a/*Δ*fet3b* (Fig. 8), which still carried a functional *fet3c* gene. These effects suggest that iron acquisition during *in vivo* infections of *M. circinelloides* depends on the amount of ferroxidases expressed by the *fet3* gene family, in which *fet3c* plays the main role, though the lack of the other two genes might be sufficient to impair iron uptake and subsequently reduce virulence potential.

In summary, we have identified a gene family of three ferroxidases with differential expression depending on the morphology of *M. circinelloides*. Our results suggest that they participate in iron acquisition when its availability is limited both *in vitro* and during *in vivo* infections. Moreover, gene *fet3c* single deletion resulted in a defect in pathogenesis, though the analysis of the double mutant strains revealed a partial redundancy in which lack of any combination of two genes is sufficient to affect virulence during *in vivo* infections. These results demonstrated the key role of this gene family of ferroxidases in the mechanism of high-affinity iron uptake of *M. circinelloides* and enlightened the crucial role of iron metabolism in mucormycosis pathogenesis.

## Materials and Methods

### Strains, growth and transformation conditions

The (−) mating type *M. circinelloides* f. *lusitanicus* CBS277.49 (syn. *Mucor racemosus* ATCC 1216b) and its derived leucine auxotroph R7B were used as the wild type strains according to their auxotrophies. Strain MU402 is a uracil and leucine auxotroph derived from R7B used for the generation of deletion mutants^31^. The *M. circinelloides* f. *lusitanicus* strain of the (+) mating type NRRL3631 was used in virulence assays as an avirulent control^13^. Cultures were grown at 26 °C in complete YPG medium or in MMC medium as described previously^31^. The pH was adjusted to 4.5 and 3.2 for mycelial and colonial growth, respectively. Transformation was carried out by electroporation of protoplasts as described previously^45^. The phenotypic growth analysis was performed in minimal media YNB pH 3.2 and iron was depleted with 50 μM of 1,10-Phenanthroline (Sigma-Aldrich). For expression analysis L15 cell culture medium (Sigma-Aldrich) was used and iron was depleted adding 10 μM of 1,10-Phenanthroline (iron-depleted medium by a synthetic iron chelator)^46^ or 20% of FBS (Sigma-Aldrich) (iron-depleted medium mimicking iron chelating in the host). In both growth and expression analyses, FeCl_3_ was added at 350 μM to obtain a high iron medium. Media were supplemented with uridine (200 mg/l) or leucine (20 mg/l) when required for auxotrophy.

### Nucleic acid manipulation and analysis

Genomic DNA from *M. circinelloides* mycelia was extracted as previously described^31^. Recombinant DNA manipulations were performed as reported^47^. Total RNA was isolated by using a RNeasy Mini kit (Qiagen, Hilden, Germany) following the supplier’s recommendations. Southern blot and northern blot hybridizations were carried out under stringent conditions^48^. DNA probes were labeled with [α-32P] dCTP using Ready-To-Go Labeling Beads (GE Healthcare Life Science). For northern blot experiments, DNA probes were directly amplified from genomic DNA using the primer pairs 187 F/187 R, 501 F/501 R and 911 F/911 R for genes *fet3a, fet3b and fet3c*, respectively (Table S2). Quantifications of signal intensities were estimated from autoradiograms using a Shimadzu CS-9000 densitometer and the ImageJ application (rsbweb.nih.gov/ij/). Differences in gene expression were analyzed by an unpaired t-test, and considered significant with a *p* < 0.01.

For Southern blot hybridizations, specific probes that discriminate between the wild type and disrupted alleles of each *fet3* gene were obtained by PCR amplification. The probe A for *fet3a* locus was obtained using the primers Dfet3aF/Dfet3apyrG (Table S2), the probe B for *fet3b* using the primers Dfet3bF/Dfet3bpyrG (Table S2), and the probe C for *fet3c* locus using the primers Ufet3cF/Ufet3cpyrG (Table S2). Markers M(Kb) was revealed adding to each probe labelling 1 ng of DNA (GeneRuler 1 kb DNA Ladder, Thermofisher).

RT-qPCR assays were performed to analyze the changes in the expression of *fet3a, fet3b* and *fet3c* genes after infection of mice with 10^6^ spores of *M. circinelloides* at day 2 post infection (n = 5). To calculate the fold-change expression, the reference sample was normalized to the expression of these genes in mycelia grown in rich medium YPG (n = 5). Total RNA was isolated by using a RNeasy Mini kit complemented with a DNase treatment (Sigma, On-Column DNaseI treatment set) and cDNA was synthesized from 1 µg of total RNA using the Expand Reverse Transcriptase for RT-qPCR (Roche). Real-time PCR was performed with a QuantStudio™ Real-Time PCR System instrument (Applied Biosystems) using 2X SYBR® Green PCR Master Mix (Applied Biosystems) and 300 nmoles of each primer (Table S2). A 10 μl reaction was set up with the following PCR conditions: Polymerase activation 95 °C for 10 min followed by 40 cycles of 95 °C for 15 sec (denature) and 60 °C for 1 min (anneal and extend). All measures were performed in triplicate and to confirm the absence of non-specific amplification, a non-template control and a melting curve were included. The efficiency of the *fet3a, fet3b* and *fet3c* amplification and the efficiency of the rRNA *18S* gen (endogenous control) amplification were approximately equal, so the relative gene expression of the above mentioned genes could be obtained using the delta-delta C_T_ (ΔΔC_T_) method normalizing for rRNA *18S*. Multiple protein sequence alignment was conducted with MUSCLE program^49^, and then curated by Gblocks to select conserved blocks of amino acids^50^. Computational phylogenetic analysis was performed using PhyML software^51^. Phylogenetic relationship was inferred by maximum likelihood-based statistical methods, employing a bootstrapping procedure of 1000 iterations.

### Virulence assays

For the murine host model, groups of 10 four-week-old OF-1 male mice (Charles River, Criffa S.A., Barcelona, Spain) weighing 30 g were used. Mice were immunosuppressed 2 days prior to the infection by intraperitoneal (i.p.) administration of 200 mg/kg of body weight of cyclophosphamide and once every 5 days thereafter. Animals were housed under standard conditions with free access to food and water. Mice were infected via tail vein with 1 × 10^6^ sporangiospores. Animals were checked twice daily for 20 days. Surviving animals at the end of the experimental period or those meeting criteria for discomfort were euthanized by CO_2_ inhalation. Mortality rate data was plotted using the Kaplan-Meier estimator (Graph Pad Prism 4.0 for Windows; GraphPad Software, San Diego California USA). Differences were considered statistically significant at a p-value of ≤0.01 in a Mantel-Cox test.

### Gene disruption

The single disruption of all *fet3* genes was achieved by double cross-over homologous recombination with disrupting cassettes generated by overlapping PCR. These cassettes were designed to contain the gene *pyrG*, which was used as a selectable marker, flanked by 1 kb upstream and downstream of the gene DNA sequences to facilitate targeted disruption by homologous recombination. To generate a specific cassette for each specific *fet3* gene, the *pyrG* selectable marker (2-kb fragment amplified from gDNA using primers pyrGF and pyrGR, Table S2) was fused with 1-kb upstream and downstream sequences of the genes *fet3a*, *fet3b* and *fet3c*, which were amplified with the primers Ufet3AF/Ufet3ApyrG and Dfet3ApyrG/Dfet3AR, Ufet3BF/Ufet3BpyrG and Dfet3BpyrG/Dfet3BR, and Ufet3CF/Ufet3CpyrG and Dfet3CpyrG/Dfet3CR (Table S2), respectively. Following this strategy, specific cassettes for each *fet3* gene were obtained and used to transform the uracil and leucine auxotrophic strain MU402 (*pyrG*−, *leuA*−), which can be complemented with the *pyrG* gene present in the cassettes (Fig. 4, upper panels), producing transformants that maintained the leucine auxotrophy. Isolates from each transformation were selected after ten rounds of sporulation and single colony isolation from selective medium MMC^31^, and screened for disruption of each *fet3* gene by PCR (Supplementary Fig. S1), using specific primer pairs in each case (Supplementary Table S2).

The double mutant Δ*fet3a*/Δ*fet3b* was obtained by gene replacement of *fet3b* with the selectable marker *leuA* in the recipient mutant strain Δ*fet3a*. The marker *leuA* was amplified with the primers leuAF/leuAR (Table S2) and fused to the adjacent sequences of the gene *fet3b* by overlapping PCR. The sequences upstream and downstream of *fet3b* were amplified with the primers Ufet3BF/Ufet3BleuA and Dfet3BleuA/Dfet3BR (Table S2). The double mutant Δ*fet3a/*Δ*fet3c* was obtained with the same strategy but with the primers Ufet3CF/Ufet3CleuA and Dfet3CleuA/Dfet3CR (Table S2) to disrupt the gene *fet3c* with the marker *leuA* in the strain Δ*fet3a*. The constructed fragment used to disrupt the gene *fet3b* with the *leuA* was also employed to obtain the double mutant Δ*fet3c/*Δ*fet3b* by transforming the recipient mutant strain Δ*fet3c*. Isolates from each transformation were selected using the same procedure described for the single mutants, except for the use of YNB instead of MMC.

### Ethics statement

All methods were performed in accordance with the regulations of Real Decreto 53/2013, of February 1st (BOE of 8 February), which is intended to assure the welfare of animals and the ethics of any procedure related to animal experimentation. Animal care procedures were supervised and approved by the Universitat Rovira i Virgili Animal Welfare and Ethics Committee. The experimental animal facilities are registered under reference T9900003 of the Generalitat de Catalunya in compliance with the regulations of Real Decreto 53/2013, of February 1st (BOE of 8 February). Procedures included into the project number 280 were supervised and approved by L. Loriente Sanz (ID 39671243) of the Veterinary and Animal Welfare Advisory of the Universitat Rovira i Virgili Animal Welfare and Ethics Committee (Reus, Spain).

## Electronic supplementary material

Supplementary Information
