# Supplementary material for: Components of a new gene family of ferroxidases involved in virulence are functionally specialized in fungal dimorphism
Source: Sci Rep. 2018 May 16;8:7660. doi: 10.1038/s41598-018-26051-x (PMC5955967; doi:10.1038/s41598-018-26051-x)
Supplement: Supplementary file 1 — Supplementary Information [file 41598_2018_26051_MOESM1_ESM.pdf]

SUPPLEMENTARY INFORMATION

Components of a new gene family of ferroxidases involved in virulence are functionally specialized in fungal dimorphism.

María Isabel Navarro-Mendoza[1], Carlos Pérez-Arques[1], Laura Murcia[1], Pablo Martínez-García[1], Carlos Lax[1], Marta Sanchis[2], Javier Capilla[2], Francisco E. Nicolás[1*] and Victoriano Garre[1*]

Affiliations:

[1] Departamento de Genética y Microbiología, Universidad de Murcia, 30100 Murcia, Spain.

[2] Unidad de Microbiología. Universitat Rovira i Virgili. IISPV. Tarragona, Spain.

Short title: Role of ferroxidases in fungal virulence and dimorphism.

*Address correspondence to:

Francisco E. Nicolás: fnicolas@um.es

Victoriano Garre: vgarre@um.es

Supplement Figure S1

Single mutants

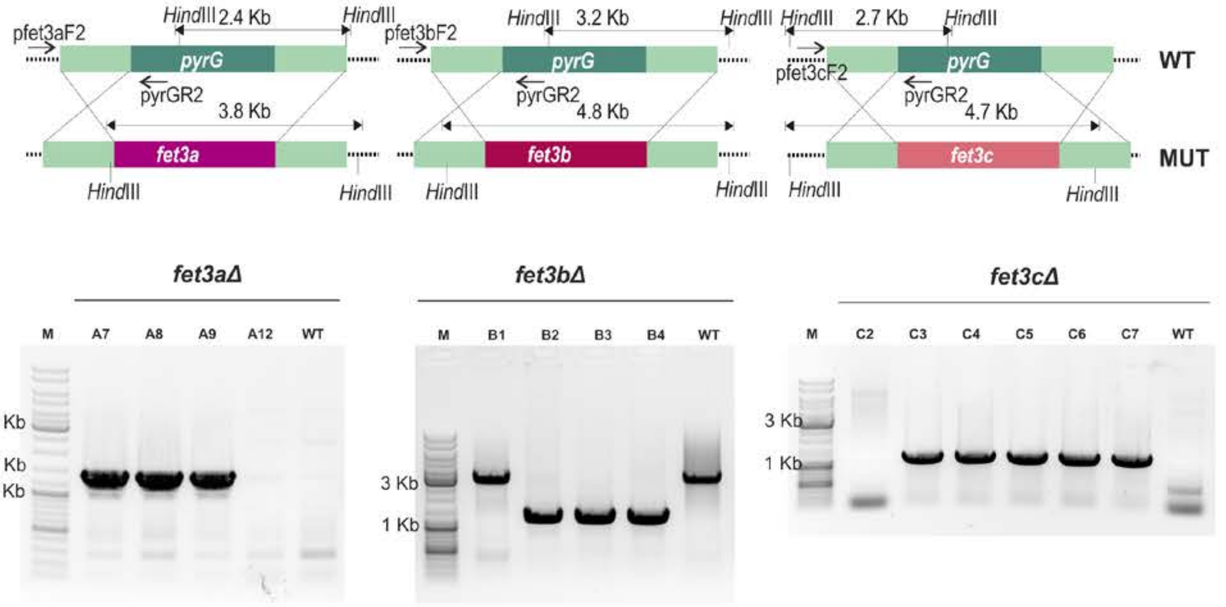

Double mutants

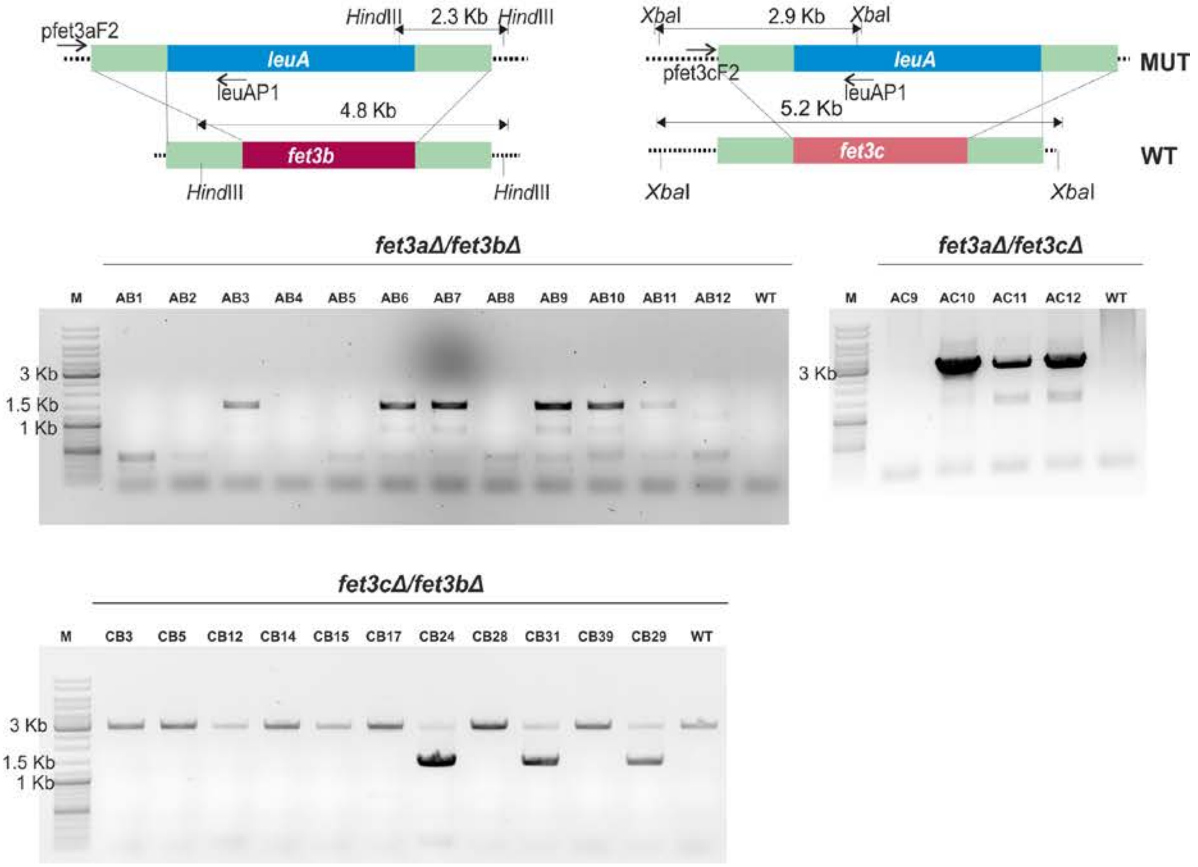

Supplementary Figure S1. PCR validation of the single and double mutants.

Gene replacement in the single deletion mutants Δ*fet3a,* Δ*fet3b and* Δ*fet3c* was validated by PCR with the following pair of primers pfet3aF2/pyrGR2, pfet3bF2/pyrGR2 and pfet3cF2/pyrGR2 (Supplementary Table S2), respectively. Genomic DNA from all the transformants obtained and the wild type strain MU402 (WT) was used as a template. The PCR product of the disrupted locus generated the expected 1.3-kb fragment in mutants Δ*fet3a*, 1.3-Kb fragment in mutants Δ*fet3b* and 1.2-Kb fragment in mutants Δ*fet3c*. Gene replacement in the double mutants Δ*fet3a*/Δ*fet3b,* and Δ*fet3c/*Δ*fet3b* was checked by PCR with the primers pfet3bF2/leuAP1 (Table S2) obtaining the expected 1.5-Kb fragment, while mutant Δ*fet3a/*Δ*fet3c* was validated with primers pfet3cF2/leuAP1 generating the expected 2.9-Kb fragment. The positions and sizes of the GeneRuler DNA ladder mixture (M) (Fermentas) are indicated.

Supplementary Figure S2. Full-length blots of Figure 3. Black dashed lines show the cropping area.

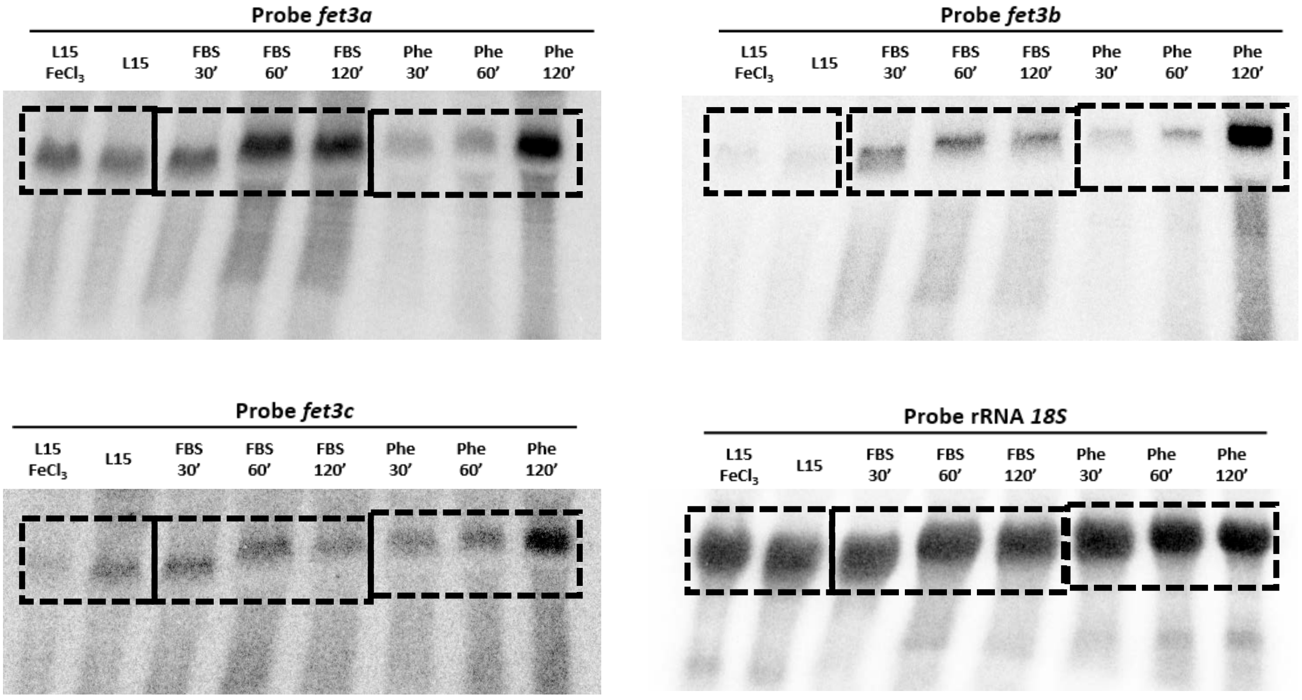

Supplementary Figure S3. Full-length blots of Figure 3. Black dashed lines show the cropping area used in Figure 4. Red dashed lines show the cropping area in Figure 5.

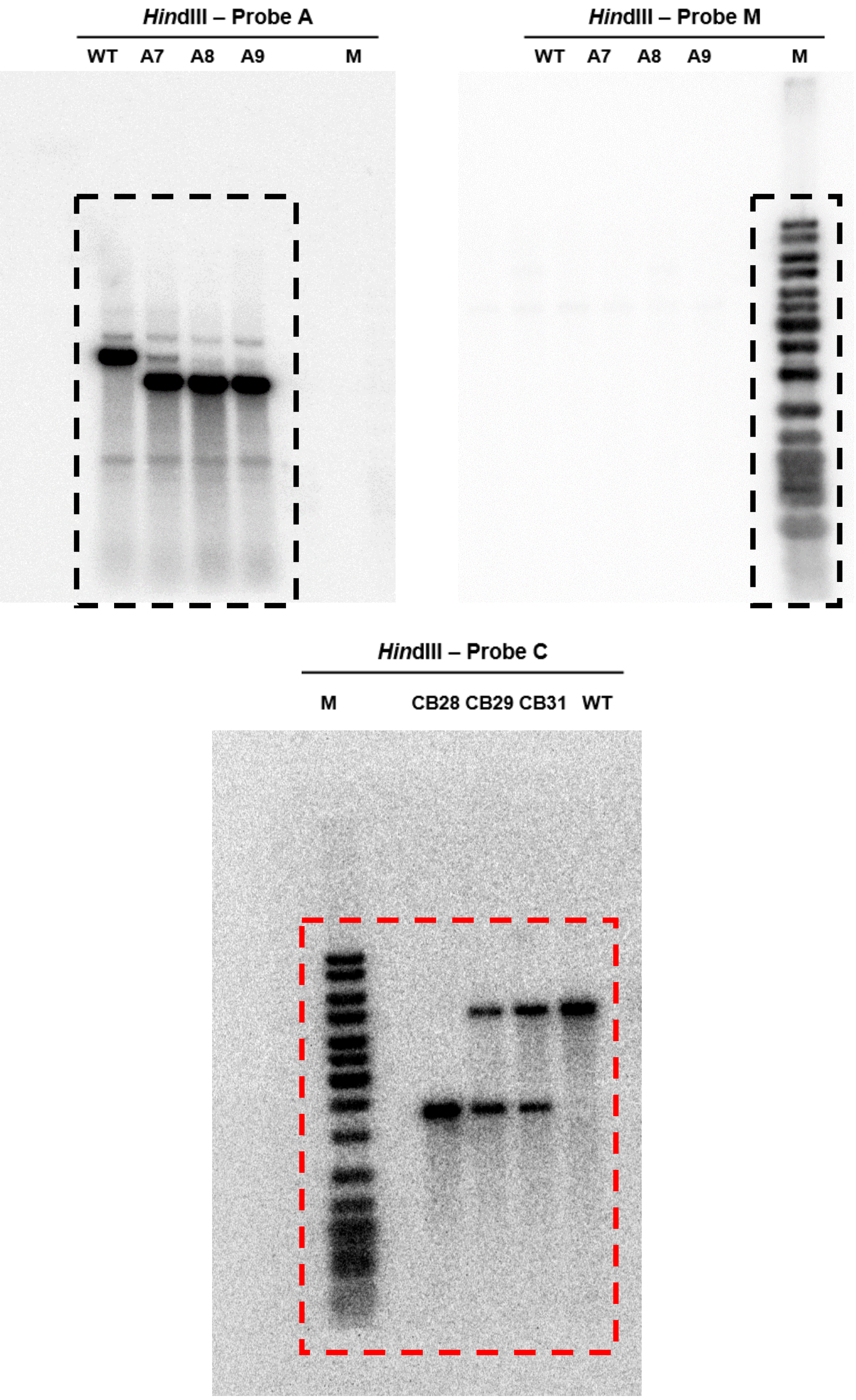

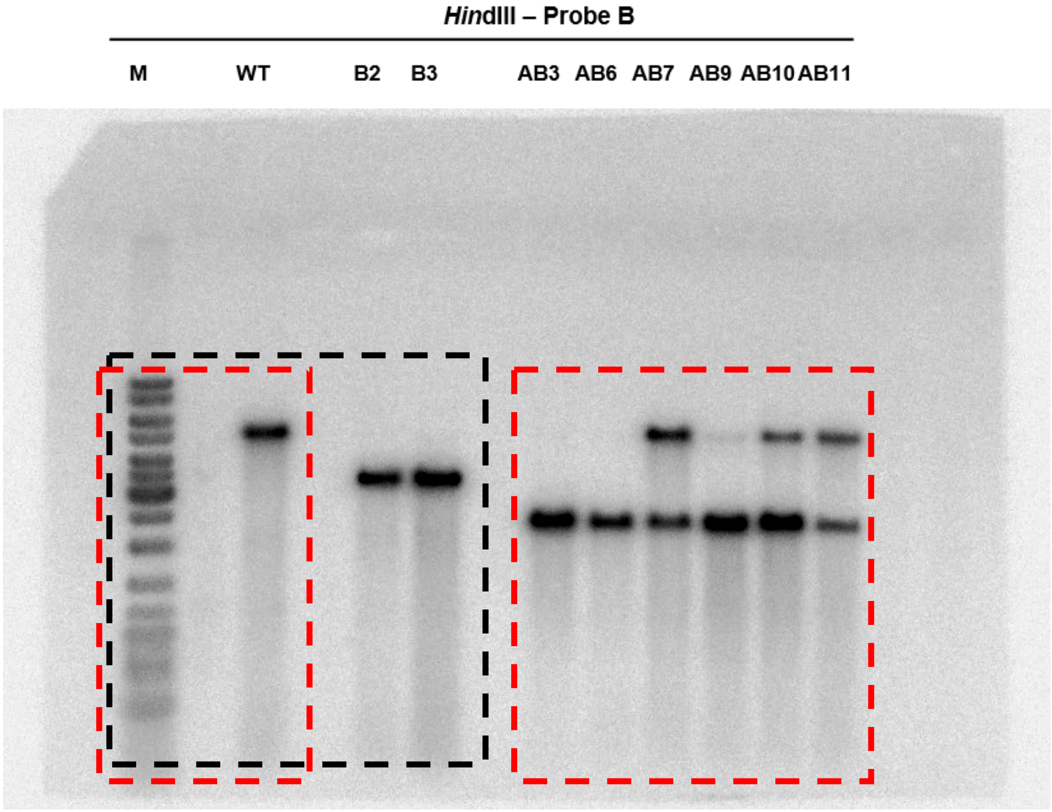
*Hin*dIII – Probe B
M
WT
B2
B3
AB3
AB6
AB7
AB9
AB10
AB11

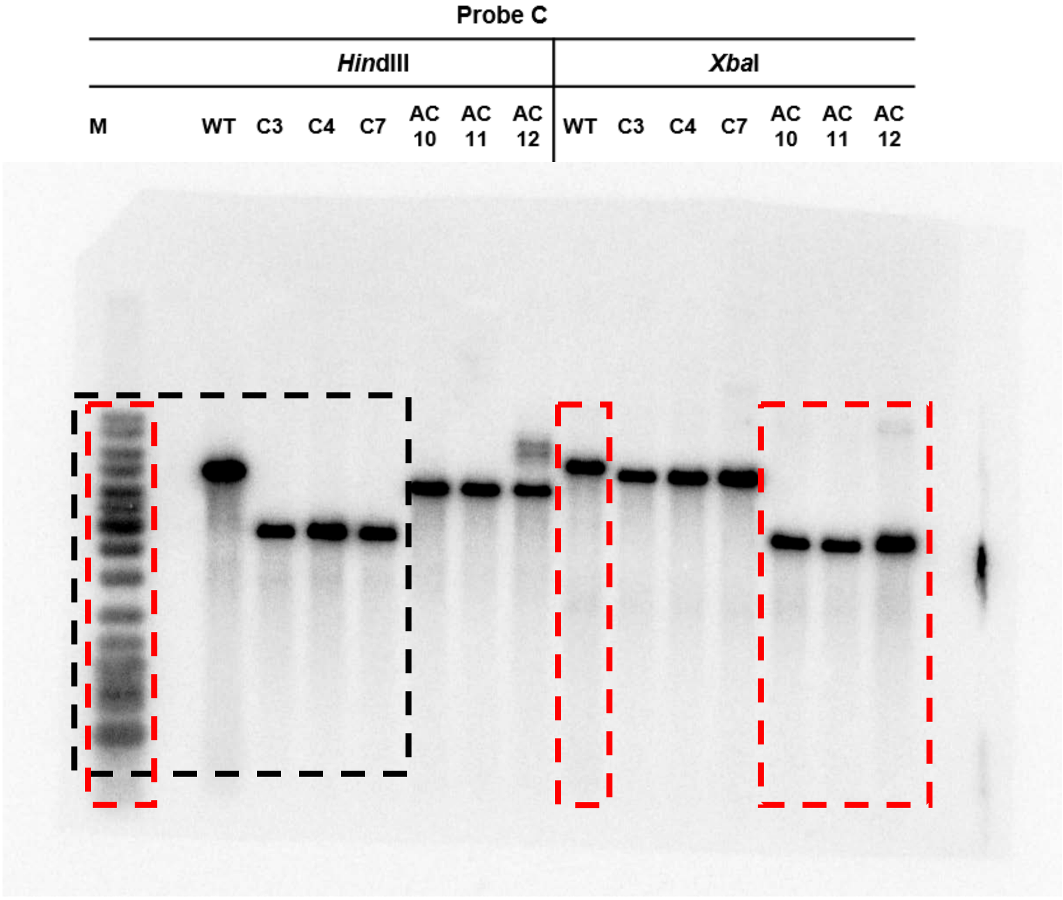
Probe C
*Hin*dIII
*Xba*I
M
WT
C3
C4
C7
AC 10
AC 11
AC 12
WT
C3
C4
C7
AC 10
AC 11
AC 12

Supplementary Figure S4. Full-length blots of Figure 7. Black dashed lines show the cropping area used.

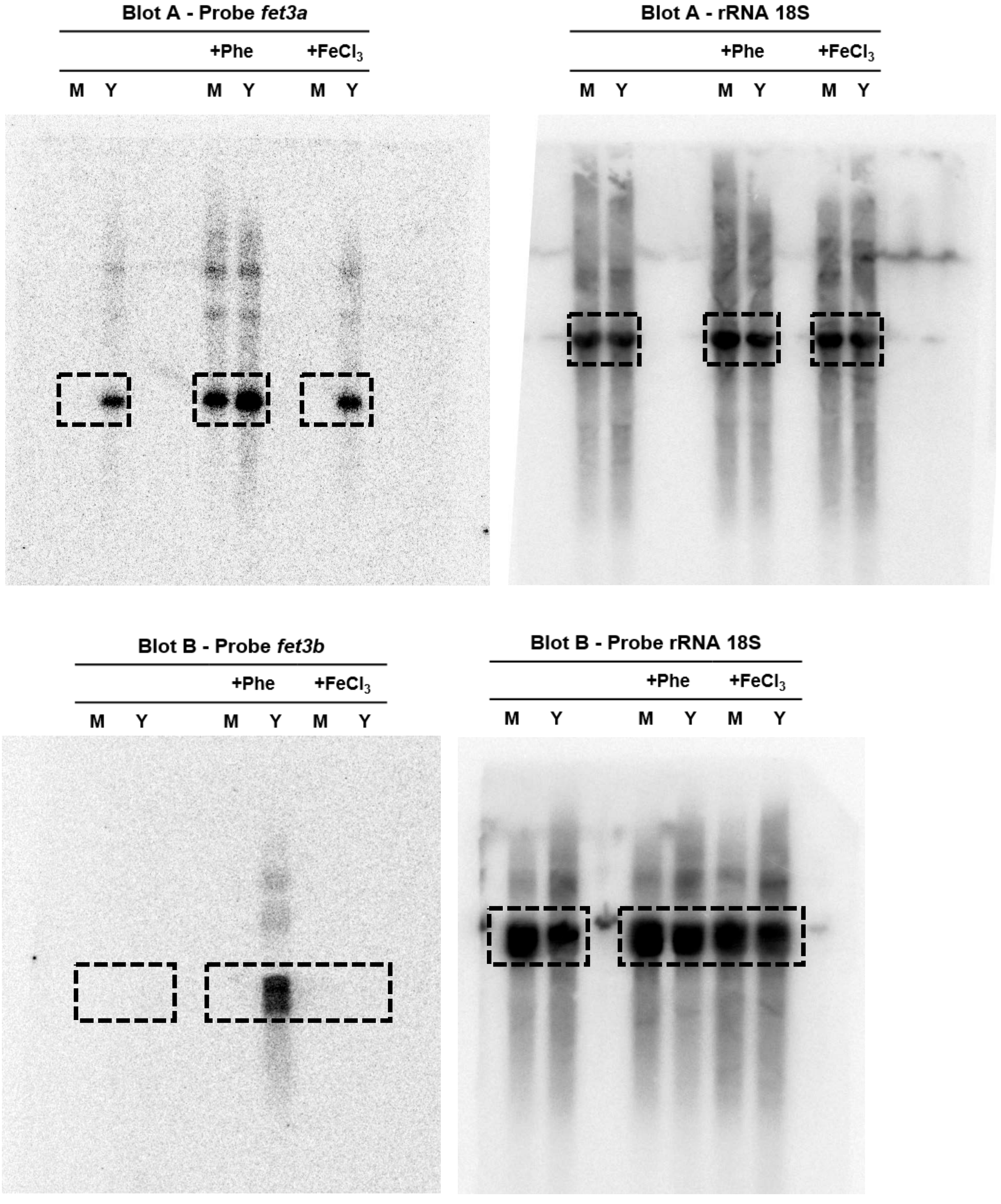

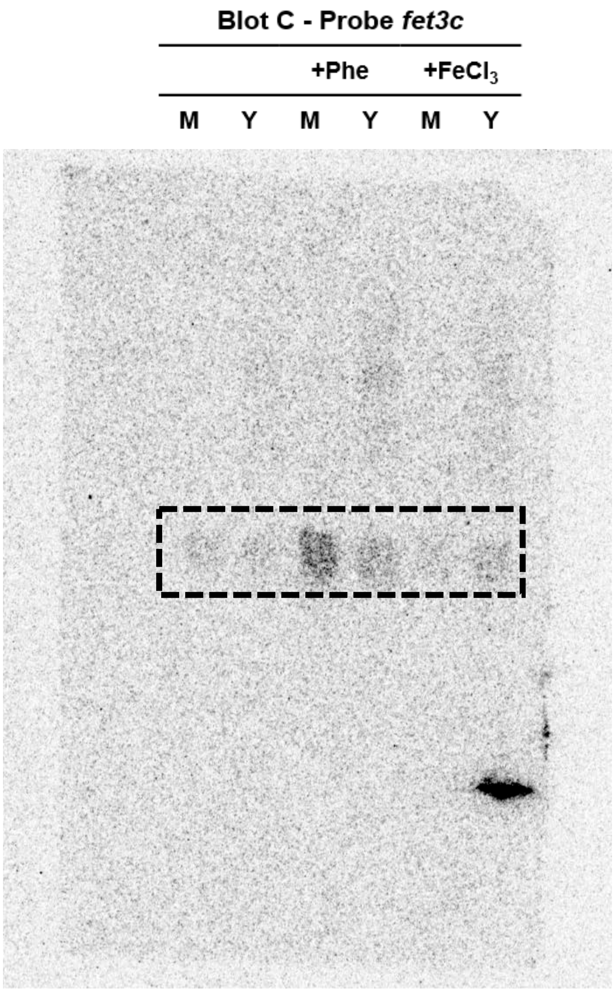
Blot C - Probe fet3c
+Phe
+FeCl3
M Y M Y M Y

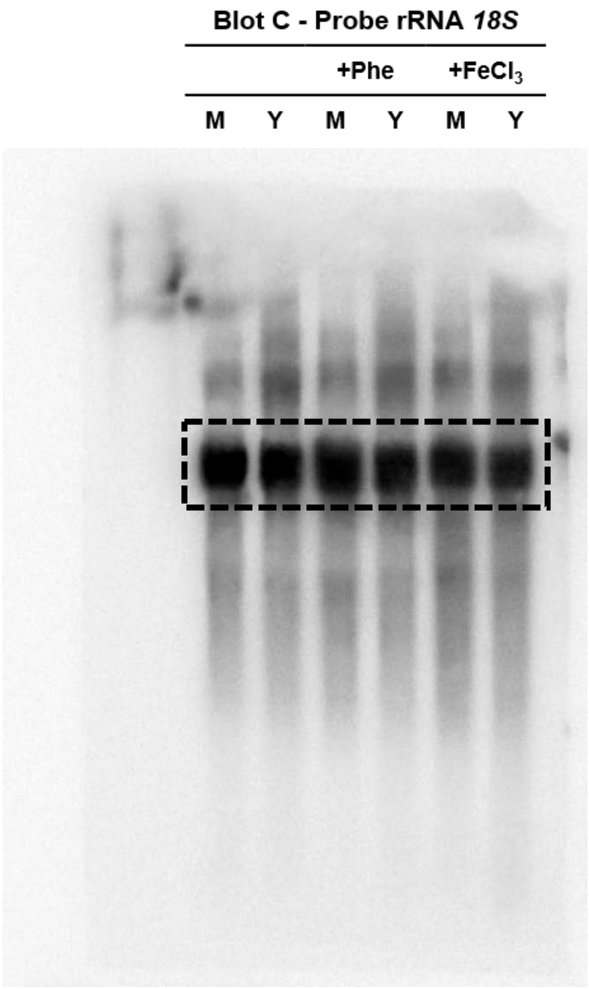
Blot C - Probe rRNA 18S
+Phe
+FeCl3
M Y M Y M Y

Supplementary Table S1. List of proteins used in the phylogenetic analysis.

	Name	ID FungiDB	ID Uniprot	ID JGI	Species	Strain	Ref. (PMID)
FERROXIDASES	ScFET3	YMR058W	P38993		*Saccharomyces cerevisiae*	S288c	8293473
	CnCfo1	CNAG_06241	J9W0J5		*Cryptococcus neoformans*	H99	19700638
	McFet3a	QYA_187130	A0A162QYZ1	187130	*Mucor circinelloides*	CBS 277.49	
	McFet3b	QYA_156933	A0A168IGP6	50174	*Mucor circinelloides*	CBS 277.49	
	McFet3c	QYA_155688	A0A168MCH1	91149	*Mucor circinelloides*	CBS 277.49	
	CaFET3	C6_00460C_A	P78591		*Candida albicans*	SC5314	10517594
	AfFetC	Afu5g03790	E9R598		*Aspergillus fumigatus*	Af293	15504822
	SpFio1	SPAC1F7.08	Q09920		*Schizosaccharomyces pombe*	972h-	8995275
	UmFer1	UMAG_00105	Q4PIF8		*Ustilago maydis*	521	17138696
LACCASES	CnLAC1	CNAG_03465	J9VY90		*Cryptococcus neoformonas*	H99	11500433
	BcLcc1	BC1G_00138	Q12570		*Botrytis cinerea*	B05.10	11929539
	NcLacc	NCU04528	P06811		*Neurospora crassa*	OR74A	2961749
	AfAbr2	Afu2g17530	E9RBR0		*Aspergillus fumigatus*	Af293	10515939

Supplementary Table S2. List of primers used in this work

NAME	SEQUENCE	USE
Ufet3aF	TGATAATGCTGTCGCCATGTGCTGG	*fet3a* disruption
pfet3aF2	CCTCCATCTCGTTCTGCAGTG	*fet3a* disruption
Ufet3apyrG	CAAGTACCAATGCTGAGGCACTCGCGATAGAATGGGTAGACCACC	*fet3a* disruption
Dfet3apyrG	CGATAGCATGGCCAGTGTACTCCATAGAATCAAAAGAGCCTGCAT	*fet3a* disruption
Dfet3aR	CACAGATGAATCCCATGATAGCAGC	*fet3a* disruption
187F	TCCTGAATTCAACATTGGCATGGCAAGCACC	Probe northern blot
187R	CTCACTCGAGACAACAGCGAGGCCTGATTCC	Probe northern blot
fet3aF	GCAGCAGTTGAGTACGACCA	RT-qPCR
fet3aR	GACCGTGAAGATGGAAAGGA	RT-qPCR
Ufet3bF	GCTTCAAGACCTTCTCTGAGGACAG	*fet3b* disruption
pfet3bF2	CGATACCCATGATAGCAGCA	*fet3b* disruption
Ufet3bpyrG	CAAGTACCAATGCTGAGGCAGAACCGGCAACTAACAGACAAGTGG	*fet3b* disruption
Ufet3bleuA	CCTTGTCCCATCCTGCAGCCTAGGCCGCCCGAACCGGCAACTAACAGACAAGTGG	*fet3b* disruption
Dfet3bpyrG	CGATAGCATGGCCAGTGTACTGAAACTGAACGGATGGCTCAGCA	*fet3b* disruption
Dfet3bleuA	GGTCAACTCGACAAATGACGCCTAGGAGAACGGCTGAAACTGAACGGATGGCTCAGCA	*fet3b* disruption
Dfet3bR	TTACCAGCACCTTTCAGTTCGACGG	*fet3b* disruption
501F	CTTTGAATTCTAGCGAAGACGTTGGCATGGG	Probe northern blot
501R	CAATCTCGAGATGGAAGAACCAAGCACCAGG	Probe northern blot
fet3bF	GTCGCGGTAACGGTGTCTAT	RT-qPCR
fet3bR	CAAAGGTTTCGGAAGGAACA	RT-qPCR
Ufet3cF	GTGCTGATCATGTAGAAGGCTTACC	*fet3c* disruption
pfet3cF2	GCTTCGCCGTCTTTGACACCCA	*fet3c* disruption
Ufet3cpyrG	CAAGTACCAATGCTGAGGCAACTGCGTATTGTTTCAACGAACAGG	*fet3c* disruption
Ufet3cleuA	CCTTGTCCCATCCTGCAGCCTAGGCCGCCCACTGCGTATTGTTTCAACGAACAGG	*fet3c* disruption
Dfet3cpyrG	CGATAGCATGGCCAGTGTACGAGCATTCAACACATTTGACAGCAG	*fet3c* disruption
Dfet3cleuA	GGTCAACTCGACAAATGACGCCTAGGAGAACGGCGAGCATTCAACACATTTGACAGCAG	*fet3c* disruption
Dfet3cR	TCAAGGATGATGTGTGTGCGTGCGT	*fet3c* disruption
911F	CCATGAATTCAGGCTCCTGCTTTGAAGGTGG	Probe northern blot
911R	CAATCTCGAGACCAAACGCCTGGATTGTCGG	Probe northern blot
fet3cF	GCCCCCATCTACTATGACGA	RT-qPCR
fet3cR	AGGAACCAGGGGAGTCAAGT	RT-qPCR
pyrGF	TGCCTCAGCATTGGTACTTG	Selectable marker amplification
pyrGR	GTACACTGGCCATGCTATCG	Selectable marker amplification
leuAF	GGGCGGCCTAGGCTGCAGGATGGGACAAGG	Selectable marker amplification
leuAR	GCCGTTCTCCTAGGCGTCATTTGTCGAGTTGACC	Selectable marker amplification
pyrGR2	ATCCCACCAGAAGGAGTACATGG	PCR validation
leuAP1	GATGTAGTTAGAGTATTTCGC	PCR validation
18SF	CCGACTAGAGATTGGGCTTG	RT-qPCR
18SR	TCTGGACCTGGTGAGTTTCC	RT-qPCR
18SF2	GGCTACCACATCCAAGGAAG	Probe northern blot
18SR2	CTCCAATTGATCCTCGTTAA	Probe northern blot

Supplementary Table S3. Strains generated in this work

Name	Genotype	Strain
MU709	*leuA-, fet3a::pyrG*	Mutant strain *Δfet3a* **A7**
MU710	*leuA-, fet3a::pyrG*	Mutant strain *Δfet3a* **A8**
MU711	*leuA-, fet3a::pyrG*	Mutant strain *Δfet3a* **A9**
MU712	*leuA-, fet3a::pyrG*	Mutant strain *Δfet3b* **B2**
MU713	*leuA-, fet3a::pyrG*	Mutant strain *Δfet3b* **B3**
MU714	*leuA-, fet3a::pyrG*	Mutant strain *Δfet3c* **C3**
MU715	*leuA-, fet3a::pyrG*	Mutant strain *Δfet3c* **C4**
MU716	*leuA-, fet3a::pyrG*	Mutant strain *Δfet3c* **C7**
MU717	*fet3a::pyrG, fet3b::leuA*	Mutant strain *Δfet3a/Δfet3b* **AB3**
MU718	*fet3a::pyrG, fet3b::leuA*	Mutant strain *Δfet3a/Δfet3b* **AB6**
MU719	*fet3a::pyrG, fet3b::leuA*	Mutant strain *Δfet3a/Δfet3b* **AB9**
MU720	*fet3a::pyrG, fet3c::leuA*	Mutant strain *Δfet3a/Δfet3c* **AC10**
MU721	*fet3a::pyrG, fet3c::leuA*	Mutant strain *Δfet3a/Δfet3c* **AC11**
MU722	*fet3a::pyrG, fet3c::leuA*	Mutant strain *Δfet3a/Δfet3c* **AC12**
MU723	*fet3c::pyrG, fet3b::leuA*	Mutant strain *Δfet3c/Δfet3b* **CB28**
